# Stoichiometry and Affinity of Thioflavin T Binding to Sup35p Amyloid Fibrils

**DOI:** 10.1371/journal.pone.0156314

**Published:** 2016-05-26

**Authors:** Anna I. Sulatskaya, Irina M. Kuznetsova, Mikhail V. Belousov, Stanislav A. Bondarev, Galina A. Zhouravleva, Konstantin K. Turoverov

**Affiliations:** 1 Laboratory for Structural Dynamics, Stability and Folding of Proteins, Institute of Cytology, Russian Academy of Science, St. Petersburg, Tikhoretsky Ave. 4, 194064, Russia; 2 Institute of Physics, Nanotechnology and Telecommunications, Peter the Great St.-Petersburg Polytechnic University, St. Petersburg, Polytechnicheskaya 29, 195251, Russia; 3 Department of Genetics and Biotechnology, Saint Petersburg State University, Saint Petersburg, Universitetskaya Emb. 7–9, 199034, Russia; 4 Laboratory of Amyloid Biology, Saint Petersburg State University, Saint Petersburg, 199034, Russia; Consejo Superior de Investigaciones Cientificas, SPAIN

## Abstract

In this work two modes of binding of the fluorescent probe thioflavin T to yeast prion protein Sup35p amyloid fibrils were revealed by absorption spectrometry of solutions prepared by equilibrium microdialysis. These binding modes exhibited significant differences in binding affinity and stoichiometry. Moreover, the absorption spectrum and the molar extinction coefficient of the dye bound in each mode were determined. The fluorescence quantum yield of the dye bound in each mode was determined via a spectrofluorimetric study of the same solutions in which the recorded fluorescence intensity was corrected for the primary inner filter effect. As previously predicted, the existence of one of the detected binding modes may be due to the incorporation of the dye into the grooves along the fiber axis perpendicular to the β-sheets of the fibrils. It was assumed that the second type of binding with higher affinity may be due to the existence of ThT binding sites that are localized to areas where amyloid fibrils are clustered.

## Introduction

Prion diseases (transmissible spongiform encephalopathies) are a class of fatal neurodegenerative diseases. Similar to other diseases associated with protein misfolding in the central nervous system, prion diseases manifest as a rapidly progressive disorder of limb reflexes, hypotension, dysarthria, swallowing disorders, ataxia and decreasing functional activity of the brain. In most cases, the disease has a sporadic origin, and less than 10% of the cases present as familial (genetic) forms: Creutzfeldt-Jakob disease, Gerstmann-Straussler-Scheinker syndrome and fatal familial insomnia [1–3]. The major neuropathological signs of transmissible spongiform encephalopathy are extensive spongiosis, gliosis [4], damage to central nervous system neurons and the deposition of amyloid plaques formed by prion proteins [5–8]; these plaques accumulate in the affected tissue, causing tissue injury and death. Prion-based amyloid fibrils are extremely stable and resistant to denaturation by chemical and physical agents (heat, SDS, urea, ultraviolet, and similar factors) [9–11]. The high mortality of prion diseases has stimulated the current intensive research in this field.

Clear evidence for prions other than the mammalian PrP protein has only been found in fungal systems; over 10 prions have been identified in *Saccharomyces cerevisiae* (see [12] for a recent review). One of the most studied prions to date is [*PSI+*], which arises as a consequence of the prionization of yeast Sup35p (translation termination factor eRF3). The Sup35 protein consists of three distinct functional domains. The C-proximal region is homologous to the translation elongation factor eEF1-A, and it is required and sufficient for translation termination and cell viability. The N-proximal region, or prion-forming domain, is uniformly rich in Gln and Asn residues and contains six oligopeptide repeats. This region is not essential for viability or termination, but it is required for [*PSI+*] induction and propagation. The charged middle (M) region is not required for viability or termination, but it can influence [*PSI+*] induction or propagation through interaction with the protein disaggregase Hsp104p (reviewed in [13]). Despite a long history of investigations (reviewed in [14]), many questions about the structural organization of Sup35p fibrils remain unresolved, which has stimulated the development of new approaches. We suggested that estimation of the parameters that characterize the binding of the benzothiazole dye thioflavin T (ThT) to amyloid fibrils may make a significant contribution to this field.

Fluorescent probes, most notably ThT, are for a long time the most widely and effectively used tools for amyloid fibrils diagnostics. ThT is used due to the specificity of its binding to amyloid fibrils and the significant change in its fluorescence quantum yield upon this interaction [15–17]. Now it became evident that examination of the parameters binding of ThT with amyloid fibrils can contribute to understanding of their formation and structure [18, 19]. Despite the large number of studies in which ThT was used to detect prion-based amyloid fibrils [1, 20–24], the mechanism underlying the interaction of this dye with these amyloid fibrils and the parameters of its binding to fibrils remain unresolved.

The majority of available data on the parameters of ThT binding to amyloid fibrils which we could find in literature are based solely on the measurements of the ThT fluorescence intensity dependence on the dye concentration in solutions containing amyloid fibrils [19, 22, 25–33]. These analyses are based on the erroneous assumptions that the recorded fluorescence intensity is proportional to the concentration of bound dye and that the fluorescence intensity plateaus when all binding sites are occupied [29]. We showed that both of these assumptions are incorrect, since the fluorescence intensity is proportional to the part of excitation light absorbed by solution, but not to the dye concentration [34]. There is another common mistake in literature regarding the determination of amyloid fibril–ThT binding parameters because fluorescence intensity per se cannot, in principle, provide information on the free dye concentration. That is why when calculating the binding parameters for many applications the concentration of bound dye was taken as the dye concentration of the injected dye that is incorrect [22, 29, 32, 35, 36].

We have shown that the correct determination of the parameters of ThT binding to amyloid fibrils can give only a spectrophotometric examination solutions prepared by equilibrium microdialysis [18]. In this work the developed approach was used to determine the parameters of ThT binding to prion protein Sup35NMp amyloid fibrils. This approach provided the first evidence that ThT binds to Sup35p amyloid fibrils through two binding modes (binding types) with significantly different binding affinities and stoichiometries; the absorption spectrum and molar extinction coefficient of the dye was also determined for each of these binding modes. The fluorescence quantum yield of the dye in each binding mode was determined using a spectrofluorimetric investigation of solutions prepared by equilibrium microdialysis, along with the subsequent correction of the recorded fluorescence intensity for the primary inner filter effect.

## Materials and Methods

### 2.1. Materials

Thioflavin T was "UltraPure Grade" from AnaSpec (USA); N-acetyl-tryptophan amide (NATA) and buffer components were from Sigma-Aldrich (USA); and ATTO-425 was from ATTO-TEC (Germany). All reagents were used without additional purification. Distilled water (for ThT) or PBS (for NATA and ATTO-425) was used as the solvent.

### 2.2. Protein expression and purification, preparation of amyloid fibril

Sup35NMp with polyhistidine tags on the N-termini were expressed in *Escherichia coli* strain BL21 host cells containing pET20b-SUP35NM [37] plasmid under T7 promotor. Overproduction of recombinant proteins was carried out in 2TYa media with 1 mM IPTG. Cultures were grown at 37°C for 6 hours. Proteins were purified in denaturing conditions (in the presence of 8 M urea) according to previously published protocols [38]. A two-step purification procedure with Ni-NTA agarose (Invitrogen) and Q-sepharose (GE Healthcare) columns was performed. Proteins were concentrated using a Centricon (30 kDa, Millipore).

To obtain aggregates of Sup35NMp, the proteins were diluted at least 100-fold in fibril assembly buffer (5 mM potassium phosphate, pH 7.4, and 150 mM NaCl) to a final protein concentration of 0.5 mg/mL. In these conditions, Sup35NMp spontaneously aggregated. Samples were incubated at 26°C with slow overhead rotation (Bio RS-24 rotator, Biosan). To monitor the amyloid fibril formation, aliquots were removed every 12 hours for up to 72 hours of incubation. The rate of protein aggregation was estimated using SDS-PAGE with boiled and unboiled samples. Sup35p amyloid fibrils are insoluble in 'cold' SDS, and upon such treatment, only monomeric proteins can enter a polyacrylamide gel.

### 2.3. Absorption measurements

The absorption spectra were recorded using a U-3900H spectrophotometer (Hitachi, Japan). For the experiments with a wide range of concentrations, Helma cells (Germany) with different optical path lengths (0.1, 0.2, 0.5, 1, and 5 cm) were used. The concentrations of ThT and Sup35p in solution were determined using molar extinction coefficients of *ε*_*412*_ = 31600 M^-1^cm^-1^ and *ε*_*276*_ = 29000 M^-1^cm^-1^ [38], respectively.

The recoded absorption spectra of ThT in the presence of amyloid fibrils (*A*(λ)) represent the superposition of the absorption spectra of free ThT, ThT bound to fibrils and the apparent absorption determined by light scattered by the fibrils (*A*_*scat*_(λ)). The dependence of apparent absorbance, determined by fibril light scattering, on *λ* is determined by equation: *A*_*ca*t_ = *aλ*^*-m*^. Coefficients *a* and *m* were determined from the linear part of the dependence *A*(λ), where there is no active dye absorption plotted in logarithmical coordinates (*lg*(*A*_*scat*_) = *f*(*lg*(λ))).

### 2.4. Fluorescence measurements

Fluorescence measurements were performed using a Cary Eclipse spectrofluorimeter (Agilent Technologies, Australia). The total fluorescence intensity $F\left(\lambda_{ex}\right)=\int_{\lambda_{em}}F\left(\lambda_{ex},\lambda_{em}\right)d\lambda_{em}$ (where *F(λ*_*ex*_,*λ*_*em*_*)* is the fluorescence intensity excited at the wavelength *λ*_*ex*_ and recorded at the wavelength *λ*_*em*_), and the fluorescence spectra were obtained using 440 nm wavelength excitation light. The spectral slit width was 5 nm for most experiments. Changing the slit widths did not influence the experimental results. The recorded total fluorescence intensity was corrected for the inner filter effect (see below and a detailed description in [34]). PBS solutions of the fluorescent dyes NATA and ATTO-425 were used as a reference to normalize the recorded and corrected values of the ThT fluorescence intensity. These values are presented as the product of the absorbance and the fluorescence quantum yield of the dye. The fluorescence quantum yield of ATTO-425 was taken as 0.9 (ATTO-TEC Catalogue 2009/2010 p. 14), and the quantum yield of NATA was taken as 0.14. All experiments were performed at room temperature (23°C).

### 2.5. Preparation of tested solutions by equilibrium microdialysis and their use to investigate the ThT-amyloid fibril interaction

Equilibrium microdialysis was performed using a Harvard Apparatus/Amika (USA) device that consists of two chambers (500 μL each) that are separated by a membrane (MWCO 10000) that is impermeable to particles larger than 10 000 Da. Sup35p amyloid fibrils in the buffer solution were placed in chamber #1; at an initial concentration *С*_*0*_, the ThT solution in the same buffer was placed in chamber #2. After equilibration, the ThT concentrations in chambers #1 and #2 become equal (*С*_*f*_), and the total ThT concentration in chamber #1 exceeded that in chamber #2 by the concentration of the bound dye (*С*_*b*_). These conditions, along with the identical volumes of chambers #1 and #2, yield the following equation:

$$C_{b}=C_{0}-2C_{f}.$$ (1)

The initial concentration of ThT in chamber #2 (*C*_*0*_) and the dye concentration in this chamber after equilibration (*C*_*f*_), which equals the concentration of free dye in chamber #1, were determined by absorption spectrophotometry. The concentration of the dye bound to fibrils (chamber #1) was determined using Eq 1. In the case of one binding mode the parameters describing the binding of ThT to amyloid fibrils could be evaluated using the following equation [39–41]:

$$C_{b}=\frac{nC_{p}C_{f}K_{b}}{1+C_{f}K_{b}}\;,$$ (2)

where *C*_*p*_ is the concentration of Sup35p at which fibrils form, *K*_*b*_ is a binding constant and ***n*** is a number of binding sites. In the case of several binding modes the following relation is valid::

$$C_{b}=\sum_{i}C_{b}{}_{i}=\sum_{i}\frac{n_{i}C_{p}C_{f}K_{bi}}{1+C_{f}K_{bi}}\;.$$ (3)

where *i* is a number of binding modes with different types of binding: different binding constants *K*_*bi*_ and number of binding sites *n*_*i*_. Taking into account that

$$A_{b}\left(\lambda \right)=\sum_{i}A_{bi}\left(\lambda \right)=\sum_{i}\varepsilon_{bi}\left(\lambda \right)C_{bi}l\;,$$ (4)

where *l* is the optical pathway length, the values of *ε*_*bi*_(λ) can be determined by multiple linear regression (e.g., using SigmaPlot) using the known values of *A*_*b*_(*λ*) and *C*_*bi*_.

When corrected for the primary inner filter effect [34], the fluorescence intensity of solutions prepared by equilibrium microdialysis *F*(*λ*_*ex*_) can be used to determine the fluorescence quantum yield of bound ThT for each binding mode (*q*_*i*_) [42]:

$$F\left(\lambda_{ex}\right)/W=F_{0}\left(\lambda_{ex}\right)=\sum_{i}A_{bi}q_{i}\;,$$ (5)

where $W=\frac{1-10^{-A_{\Sigma }}}{A_{\Sigma }}$ is a correction factor, and *A*_Σ_ = ∑ *A*_*bi*_ + *A*_*f*_ is the total absorbance of the solution determined by the absorbance of the dye in the free form (*A*_*f*_) and the form bound to fibrils (∑ *A*_*bi*_).

### 2.6. Electron microscopy

To obtain electron micrographs an electron microscope Libra 120 (Carl Zeiss, Germany) and the method of negative staining with a 1% aqueous solution of uranyl acetate were used. Amyloid fibrils were placed on copper grids coated with a collodion film-substrate.

## Results and Discussion

The ThT interaction with Sup35p amyloid fibrils was investigated using solutions prepared by equilibrium microdialysis, as described in "Materials and Methods". This preparation method is essential because in solutions of ThT and fibrils, the free dye is in equilibrium with the dye bound to fibrils, and only the proposed approach allows the quantification of the free and bound ThT fractions [43].

### The absorption spectrum of ThT bound to Sup35p amyloid fibrils

**Fig 1:**
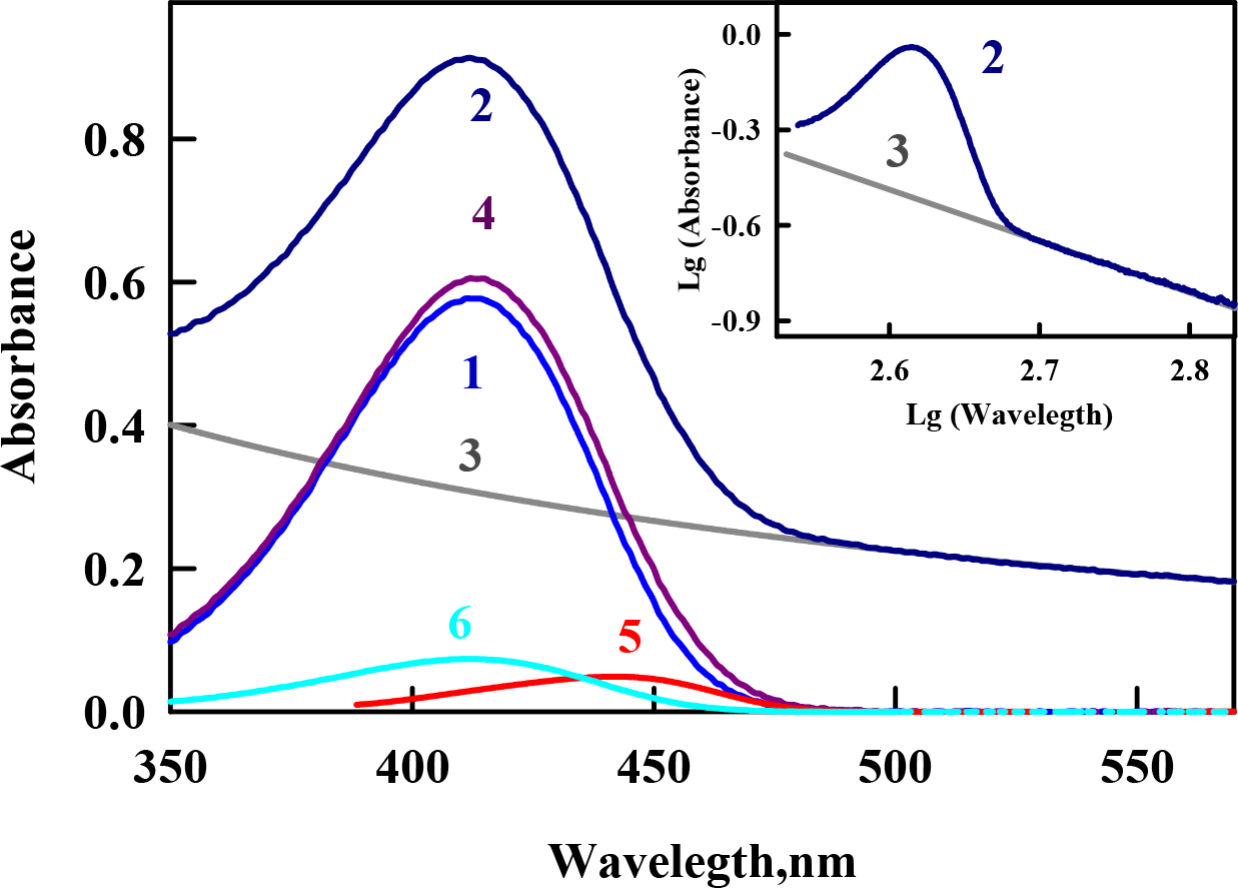
Absorption spectra of thioflavin T (ThT) incorporated into Sup35p amyloid fibrils. Curves 1 and 2 represent the absorption spectra of ThT in chamber #1 (free ThT at concentration *C*_*f*_) and in chamber #2 (superposition of the absorption spectra of free ThT at concentration *C*_*f*_, ThT bound to fibrils at concentration *C*_*b*_, and the apparent absorption caused by the light scattering) after equilibrium was attained. Curve 3 represents the optical density caused by the fibril light scattering as calculated by the equation *A*_*scat*_ *= aλ*^*-m*^. The coefficients *a* and *m* were determined from the linear part of curve 2 (where there is no active dye absorption) plotted in logarithmic coordinates *lg(A*_*scat*_*) = f(lg(λ))* (see Insert, curve 3). Curve 4 represents the total absorption of free and bound dye after light scattering subtraction (*A(λ)#2 –D*_*scat*_). Curve 5 is the absorption spectrum of ThT incorporated in the amyloid fibrils evaluated as *A*_*b*_*(λ) = A(λ)#2 –A(λ)*_*scat*_*−A(λ)#1* (the difference between spectra 4 and 1). Curve 6 is the absorption spectrum of the free dye at a concentration equal to that of bound dye (*A(λ)0 – 2A(λ)#1*).

The equilibrium microdialysis was repeated several times using different input concentrations of ThT (*C*_*0*_). As a result, a large set of solutions with different ThT concentrations was prepared, and the absorption spectrum was determined for each solution. The contribution of the light scattering of the amyloid fibrils to the recoded spectra was eliminated as described earlier [44]. Using the solutions prepared by equilibrium microdialysis provided the first measurement of the absorption spectrum of ThT bound to Sup35p fibrils (Fig 1). The significantly shorter wavelength position of the absorption spectrum of free ThT in solution (λ_max_ = 413 nm) compared with that of ThT incorporated into Sup35p amyloid fibrils (λ_max_ = 440 nm) can be explained by the oriented dipole–dipole interaction of the dye molecules with a polar solvent. The ground state of ThT in aqueous solution is stabilized by the orientational interactions of the polar solvent dipoles with the dipole caused by the positive charge of the ThT molecule, which is unequally distributed between the benzothiazole and aminobenzoyl rings. In contrast, the configuration of the solvation shell of the ThT molecule in the excited Franck-Condon state is far from an equilibrium state (see [44, 45]). Therefore, the ThT absorption spectrum has the shortest wavelength maximum in water (413 nm), and the absorption spectra of ThT bound to amyloid fibrils are red-shifted. Previous studies demonstrated that the maximal shift in the absorption spectrum is observed for the dye bound to insulin- (λ_max_ = 450 nm) and lysozyme-based (λ_max_ = 449 nm) amyloid fibrils [18, 39, 43], whereas the shift for ThT bound to Sup35p amyloid fibrils is the same as in the case of Abeta-peptide fibrils (λ_max_ = 440 nm). The position of the absorption spectrum of ThT incorporated into amyloid fibrils can differ depending on the proteins and peptides that form the fibrils because of the different microenvironments of the bound dye molecules.

### The affinity and stoichiometry of ThT binding to Sup35p amyloid fibrils

**Fig 2:**
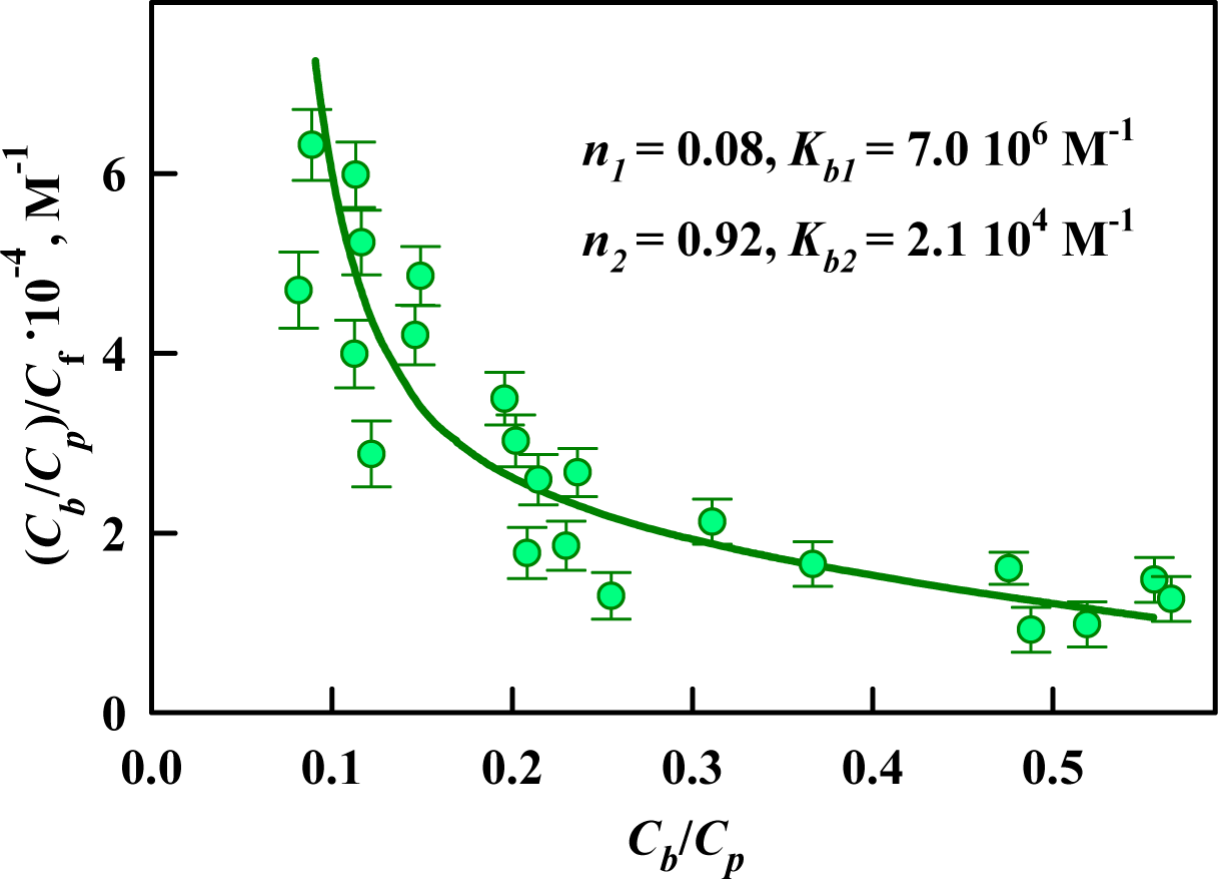
Scatchard plot for the ThT interaction with Sup35p amyloid fibrils. The experimental data (circles) and best fit curve with the binding constants (*K*_*bi*_) and the number of binding sites (*n*_*i*_) are shown.

The obtained absorption spectra were used to calculate the concentrations of the dye in the free (*C*_*f*_) and fibril-bound (*C*_*b*_). The results are presented using Scatchard coordinates (Fig 2):

$$\left(\frac{C_{b}}{C_{p}}\right)/C_{f}=nK_{b}-K_{b}\left(\frac{C_{b}}{C_{p}}\right).$$ (6)

This representation of the experimental results has the advantage of providing a visualization of the number of modes of binding between the ligand and the receptor (different binding types). A linear character of this dependence may indicate the identity and independence of all of the existing binding sites. Non-linearity of the Scatchard plot, as in the case of the ThT interaction with Sup35p amyloid fibrils (Fig 2), indicates the presence of two or more different binding modes with different affinities between the ligand and the receptor. The binding constants (*K*_*b1*_ and *K*_*b2*_) and number of binding sites of ThT to fibrils per protein molecule (*n*_*1*_ and *n*_*2*_) were determined using Eq 2 assuming the existence of two binding modes (*i* = 2). The experimental data were satisfactorily approximated by the calculated curve, which was plotted using the obtained values of the binding parameters; this correspondence demonstrates the correctness of the chosen binding model and the determined binding parameters.

To estimate the locations in which the detected binding modes occur, the existing notions about the packing of Sup35p monomers in the fibrillar structure and the parameters of ThT binding to the amyloid fibrils formed by other amyloidogenic proteins and peptides were analyzed. The N-domains of Sup35p that form the amyloid fibrils are stacked in a parallel super-β-sheet structure and linked together by hydrogen bonds [46]. Thus, the β-sheets are oriented perpendicular to the axis of the fibrils and thereby form a stack of planar molecules. We believe that one of the detected binding modes (the mode with lower affinity and a larger number of binding sites) is caused by the incorporation of ThT molecules into the grooves formed by the side chains of amino acids along the long axis of the fibril perpendicular to the fibril β-sheets. This type of dye binding to amyloid fibrils was predicted by Krebs [47] and was subsequently confirmed in other studies [48]. We believe that this type of binding is characterized by a lower binding constant (~ 10^4^ M^-1^) because the mode with this affinity is observed for different amyloid fibrils such as insulin, lysozyme [18, 39, 43] and beta-2-microglobulin [49] amyloid fibrils (Table 1), which is consistent with the views of their overall architecture. Since the N-domain of Sup35p contains sufficient β-sheets for ThT binding (size of approximately 16 Å), one would expect a 1/1 stoichiometry of the dye binding per protein molecule. The number of ThT binding sites per protein molecule for this mode is actually less than 1 (9 ThT molecules correspond to 10 Sup35p molecules); this discrepancy may occur because some binding sites are located in the interior of the fibril and are inaccessible to the dye molecules and also because some sites in the fibril grooves may participate in the other binding mode.

**Table 1:** Characteristics of thioflavin t bound to amyloid fibrils and free dye in aqueous solution.

Object	mode	λ_max_, nm	*ε*_*i*, max_x10^-4^, M^-1^cm^-1^	*K*_*bi*_ x10^-5^, M^-1^	n_i_	q_i_
Sup35p fibrils	1	440	1.8±0.3	71±10	0.08±0.08	0.24±0.04
	2	446	1.4±0.1	0.21±0.17	0.92±0.20	0.08±0.03
Lysozyme fibrils [39, 42]	1	448	5.1±0.4	75±11	0.11±0.02	0.44±0.05
	2	449	6.7±0.6	0.56±0.08	0.24±0.03	(5±5)·10^−4^
Insulin fibrils [18, 43]	1	449	8.7±0.6	200±120	0.01±0.01	0.83±0.05
	2	451	3.5±0.3	1.2±0.9	0.08±0.03	0.30±0.03
Beta-2-microglobulin fibrils [49]	1	430	4.2±0.3	0.14±0.07	0.02±0.01	0.07±0.04
Free in aqueous solution [52]	-	412	3.2±0.1	-	-	(1±1)·10^−4^

**Fig 3:**
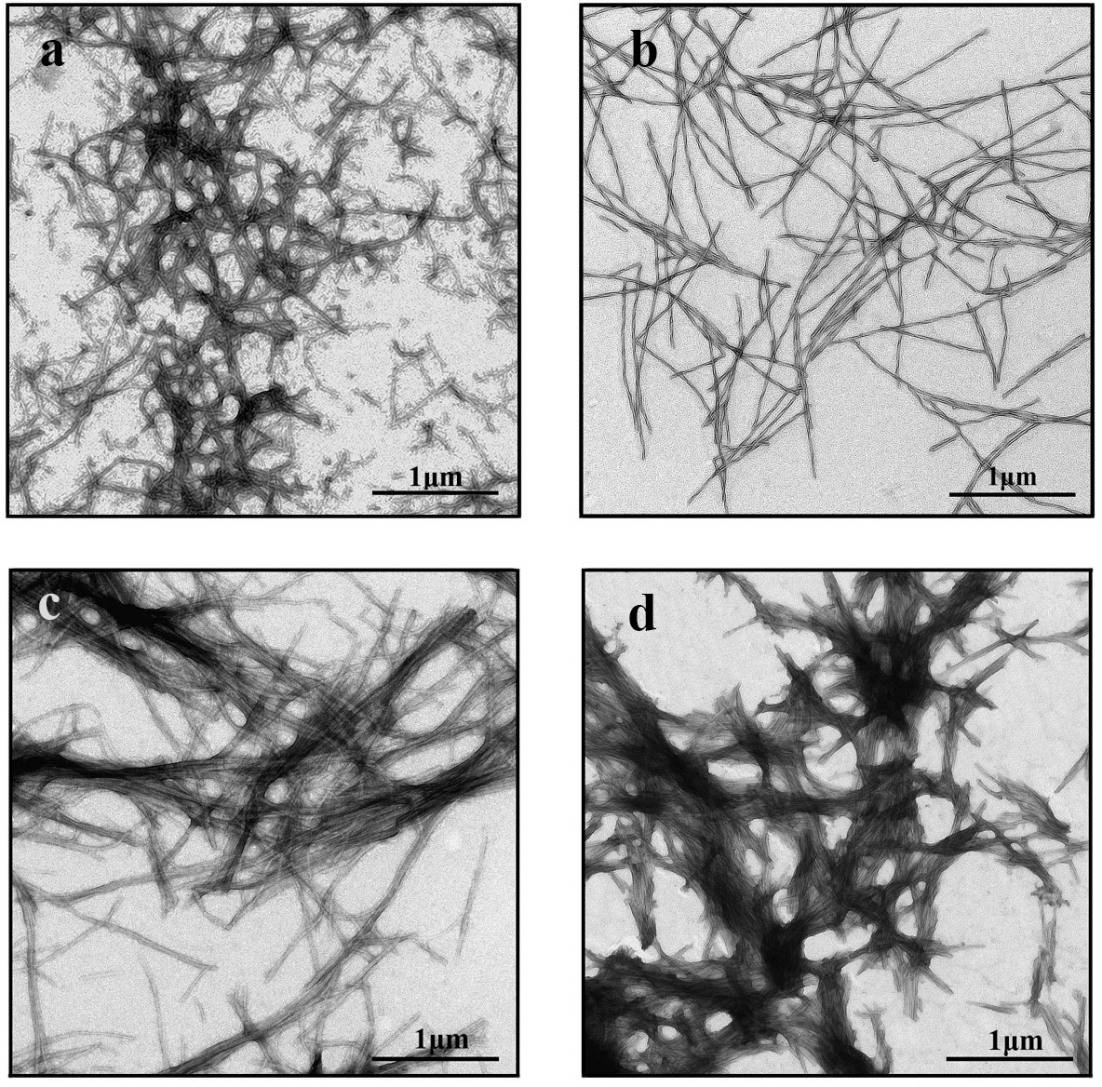
Electron micrographs. **(**a) Sup35p, (b) beta-2—microglobulin, (c) insulin, and (d) lysozyme amyloid fibrils.

It is interesting that the binding mode with higher affinity (~ 10^6^ M^-1^), which was detected in the investigated Sup35p amyloid fibrils, has not been observed in some other fibrils such as beta-2-microglobulin [49] amyloid fibrils (Table 1). We showed that the fibrils that have a single mode of ThT binding are separate long thin fibers without areas of aggregation (e.g., fibrils based on beta-2-microglobulin, shown in Fig 3B), whereas the fibrils with two ThT binding modes have a propensity to cluster (e.g., fibrils based on Sup35p, insulin or lysozyme, as shown in Fig 3A, 3C and 3D, respectively). It can be assumed that existence of the modes with a high binding affinity is due to the incorporation ThT in areas of amyloid fibrils aggregation. We believe that the localization of dye molecules in these fibril clusters (possibly within the above-discussed grooves along the fiber axis) causes the high binding affinity because of the strong attachment of the ThT molecule in this interaction. The relatively low number of binding sites that participate in this mode (1 ThT molecule corresponds to 10 protein molecules) may be due to the inaccessibility of these binding sites and the low degree of clustering of the investigated amyloid fibrils.

### The molar extinction coefficient of ThT bound to Sup35p amyloid fibrils

**Fig 4:**
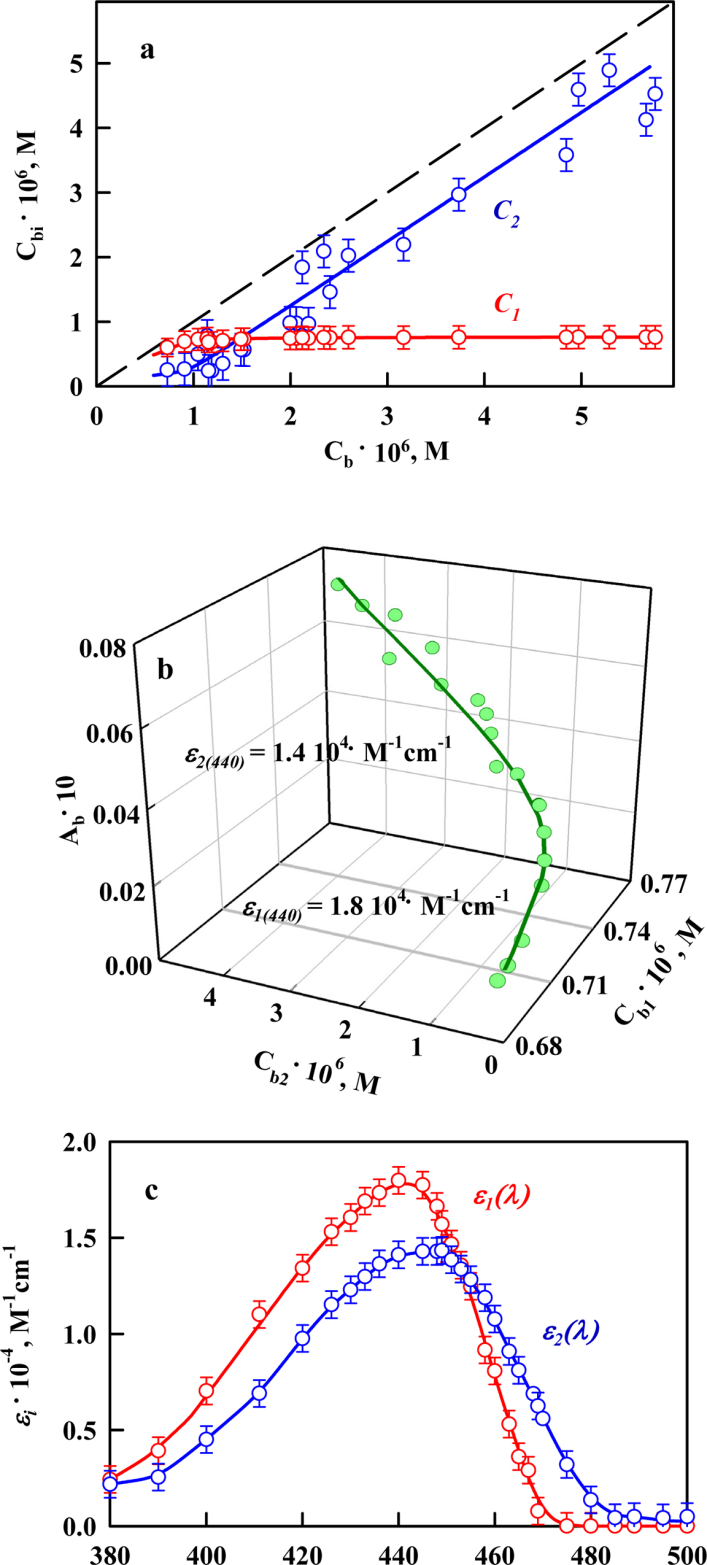
Determination of the molar extinction coefficient of ThT bound to Sup35p amyloid fibrils. **(a)** Concentration of ThT bound to amyloid fibrils (*C*_*b*_) as a superposition of the concentrations of dye bound to mode 1 (*C*_*b1*_) and mode 2 (*C*_*b2*_). **(b)** 3D dependence of *A*_*b*_ = *A*_*b*1_ + *A*_*b*2_ = *ε*_*b*1_*C*_*b*1_*l* + *ε*_*b*2_*C*_*b*2_*l* on *C*_*b1*_ and *C*_*b2*_. The experimental data and the values of the molar extinction coefficients *ε*_*b1*_ and *ε*_*b2*_ obtained by multiple nonlinear regression are presented. **(c)** Absorption spectra of ThT for mode 1 and mode 2 of the binding to Sup35p amyloid fibrils in units of the molar extinction coefficient.

For each microdialysis experiment, the concentration of ThT for different modes of binding to Sup35p amyloid fibrils was calculated using Eq 3 (Fig 4A). Then, using Eq 4, the molar extinction coefficients of ThT associated with different binding sites were determined (Fig 4B). Fig 4C shows the absorption spectra in units of molar extinction coefficient for the dye in the binding sites participating in each binding mode with Sup35p amyloid fibrils. These results indicate that the interaction of ThT with Sup35p fibrils via different binding modes leads to a slight decrease in the molar extinction coefficient of ThT relative to that of the free dye in aqueous solution; this decrease was also observed for ThT bound to the amyloid fibrils formed by other proteins (Table 1) [18, 39, 43]. An increase or decrease in the molar extinction coefficient of a dye when it binds to fibrils may be explained using quantum chemical calculations. These calculations predicted that the oscillator strength for the ThT transition from the ground state to the excited state will depend on the *φ* angle value between the benzothiazole and aminobenzoyl rings [50]. Thus, it can be assumed that when ThT interacts with Sup35p amyloid fibrils, the conformation of the dye molecule can be changed, consequently changing the angle between benzothiazole and aminobenzoyl rings of this molecular rotor from *φ* = 37°, which is the typical value for the free dye in aqueous solution [44].

### The fluorescence quantum yield of ThT bound to Sup35p amyloid fibrils

To determine the fluorescence quantum yield of ThT for each binding mode with Sup35p amyloid fibrils, solutions prepared by equilibrium microdialysis were investigated by fluorescence spectroscopy. When we analyzed the dependence of the recorded fluorescence intensity of fibril-associated ThT on its absorption, the problem of the inner filter effect was solved. The fluorescence intensity was measured using a Cary Eclipse spectrofluorimeter with a horizontal configuration of slits [34]; therefore, analytically calculated correction factors were used to correct the recorded fluorescence intensity values (see. Materials and Methods); the correction factors are determined using only the total absorption of the investigated solutions.

**Fig 5:**
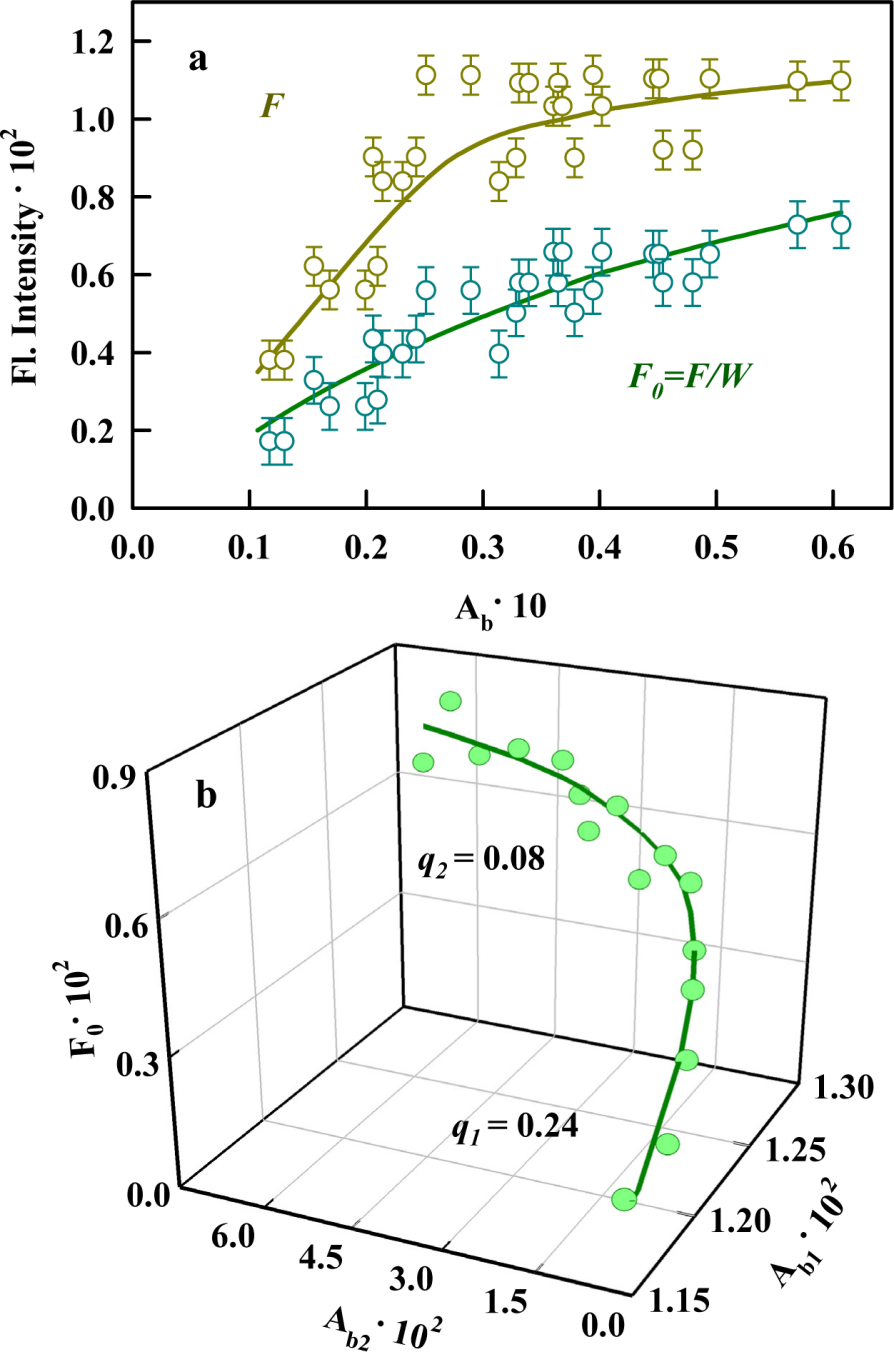
Determination of the fluorescence quantum yield of ThT bound to Sup35p amyloid fibrils. **(a)** The dependencies of the experimentally recorded fluorescence intensity (*F*) and corrected fluorescence intensity (*F*_*0*_ *= F/W*) of ThT bound to fibrils on its absorption (*A*_*b*_). Fluorescence measurements were conducted using a Cary Eclipse spectrofluorimeter (Varian, Australia). **(b)** 3D dependence of *F*_0_ = *A*_*b*1_*q*_*b*1_ + *A*_*b*2_*q*_*b*2_ on *A*_*b1*_ and *A*_*b2*_. The experimental data and the values of the fluorescence quantum yields *q*_*b1*_ and *q*_*b2*_ obtained by multiple nonlinear regression are presented.

If the straight line intersecting the X- and Y-axes at the origin provides a satisfactory approximation of the dependence of the ligand fluorescence intensity on its absorption when the fluorescence is corrected for the primary inner filter effect, it is possible that just one type of fluorescent center exists. This situation is possible in the following circumstances: when only a single binding mode exists; when several binding modes exist, but molecules only fluoresce in one of the binding modes (the other molecules undergo a nonradiative transition to the ground state); and when the fluorescence quantum yields of the ligand bound in various modes coincide. The dependence of the fluorescence intensity of ThT bound to Sup35p amyloid fibrils on its absorption (presence of the sections with different slopes) (Fig 5A) was nonlinear, confirming our assumption that several binding modes exist for the dye and the investigated fibrils and suggesting that there are differences in fluorescence quantum yield for the dye bound via each of these. Thus, the fluorescence quantum yield of the dye bound to Sup35p amyloid fibrils (Fig 5B) was determined using the assumption that two separate absorbing and fluorescing centers (*i* = 2) exist and using Eq 5.

The obtained values of the fluorescence quantum yield of ThT bound to fibrils significantly exceed the value for the free dye in aqueous solution (Table 1). Using the analysis of the ThT photophysical properties in solutions of different viscosities (water-glycerol mixtures at different temperatures), we have shown that the value of the fluorescence quantum yield of the dye may be governed by the mobility of the benzothiazole and aminobenzene rings relative to one another in the excited state and by the conformation of the molecules in the ground state [51]. It should be noted that the ThT molecules bound to the sites with higher affinity have a fluorescence quantum yield (Table 1) similar to the value for the dye in a rigid isotropic solution (q = 0.28) [52]. This similarity may be due to the rigidity of the microenvironment when dye molecules are located deep within fibril clusters. At the same time, the molecules that are incorporated into the grooves of amyloid fibrils have a fluorescence quantum yield that is approximately 2-fold less (Table 1), indicating that these molecular regions remain mobile relative to one another in the excited state.

## Conclusion

In this paper, the absorption spectroscopy of solutions prepared by equilibrium microdialysis was used to obtain the first correct evaluation of the parameters characterizing the binding of the fluorescent probe thioflavin T (ThT) to amyloid fibrils of the yeast protein Sup35p. Two modes of binding were shown between the dye and fibrils; these binding modes exhibit significant differences in affinity and binding stoichiometry, as well as in the fluorescence quantum yield of the bound dye. The proposed approach is essential because the widely used method of determining the ThT-fibril binding parameters, that is, measuring the dependence of the fluorescence intensity of ThT-fibril solutions on the dye concentration, cannot be used when several binding modes exist. Therefore, the common method cannot provide accurate results for the interaction of ThT with Sup35p fibrils.

One of the detected binding modes was previously predicted, and its existence may be due to the incorporation of the dye into the grooves along the fiber axis perpendicular to the fibril β-sheets. The presence of an additional type of binding with higher affinity, the smaller number of binding sites and the higher fluorescence quantum yield of the fibril-bound ThT can be caused by the localization of the dye molecules in fibril clusters (possibly within the same grooves along the axis of the fibrils). The difference in the dye binding parameters depending on the sites of the observed modes and the characteristics of the bound dye can be caused by the following factors: different availabilities of the sites of these modes and their different quantities (determines the different number of binding sites to these modes per protein molecule) and the different rigidities of the microenvironment of the bound molecule (defines the differences in the binding constant and the fluorescence quantum yield of ThT).

For the diseases that accompany various types of amyloidosis (including prion diseases), the formation of individual fibril strands is observed; these strands are not evenly distributed throughout the body and are clustered into insoluble deposits in organs and tissues. We must pay particular attention to this fact because of the wide use of ThT as a diagnostic and possibly therapeutic agent for these diseases. We can assume that in the bodies of patients with amyloidosis, the interaction of ThT, its derivatives and its analogs with clusters and aggregates of amyloid fibrils can dominate over the interaction with individual fine filaments. Hence, in *in vitro* experiments, special attention should be given to the characterization of this type of interaction.
